# Construction of Electrochemical Sensors for Antibiotic Detection Based on Carbon Nanocomposites

**DOI:** 10.3390/nano12162789

**Published:** 2022-08-14

**Authors:** Aihemaitijiang Aihaiti, Zongda Li, Yanan Qin, Fanxing Meng, Xinbo Li, Zekun Huangfu, Keping Chen, Minwei Zhang

**Affiliations:** 1 College of Life Science & Technology, Xinjiang University, Urumqi 830017, China; 2 Xinjiang Key Laboratory of Biological Resources and Genetic Engineering, Urumqi 830017, China; 3 Xinjiang Huize Foodstuff Co., Ltd., Wujiaqu City 830073, China

**Keywords:** antibiotics, graphene, carbon nanotubes, functionalization, electrochemical activity, aptamers, molecular imprinting

## Abstract

Excessive antibiotic residues in food can cause detrimental effects on human health. The establishment of rapid, sensitive, selective, and reliable methods for the detection of antibiotics is highly in demand. With the inherent advantages of high sensitivity, rapid analysis time, and facile miniaturization, the electrochemical sensors have great potential in the detection of antibiotics. The electrochemical platforms comprising carbon nanomaterials (CNMs) have been proposed to detect antibiotic residues. Notably, with the introduction of functional CNMs, the performance of electrochemical sensors can be bolstered. This review first presents the significance of functional CNMs in the detection of antibiotics. Subsequently, we provide an overview of the applications for detection by enhancing the electrochemical behaviour of the antibiotic, as well as a brief overview of the application of recognition elements to detect antibiotics. Finally, the trend and the current challenges of electrochemical sensors based on CNMs in the detection of antibiotics is outlined.

## 1. Introduction

Antibiotics are chemicals that inhibit the growth of bacteria and even kill them. Since their discovery, antibiotics have considerably improved human and animal health. However, the abuse of antibiotics favours their presence in animals and food, leading to allergies, antibiotic resistance, and superinfections in humans [1,2,3,4,5,6,7,8,9]. Therefore, sensitive and selective methods are required to detect antibiotics. At present, numerous analytical methods have been constructed for the detection of antibiotics, such as high-performance liquid chromatography (HPLC), enzyme-linked immunosorbent assay (ELISA), capillary electrophoresis (CE), and liquid chromatography–mass spectrometry (LC–MS) [10,11,12,13,14,15,16]. Although these analytical methods exhibit high sensitivity and a low detection limit, they still have the disadvantages of expensive instruments and complex pretreatment processes.

Nowadays, rapid methods for the detection of antibiotics include colorimetric, fluorescent, and electrochemical methods [17,18,19,20]. Compared to these, electrochemical methods are attracting attention for their high sensitivity, high accuracy, and trace detection. However, traditional electrode materials have obvious drawbacks, such as the high potential value required for the electro-oxidation of these compounds [21,22,23,24]. Due to their excellent electrical conductivity, large surface area, and high electronic transport, the use of carbon nanomaterials (CNMs), such as graphene (Gr) and its derivatives, including graphene oxide (GO) and reduced graphene oxide (rGO), as well as carbon nanotube (CNT), including multi-walled carbon nanotube (MWCNT) and single-walled carbon nanotube (SWCNT), has been an effective strategy that has been gaining attention [25,26,27,28,29,30,31,32].

**Figure 1:**
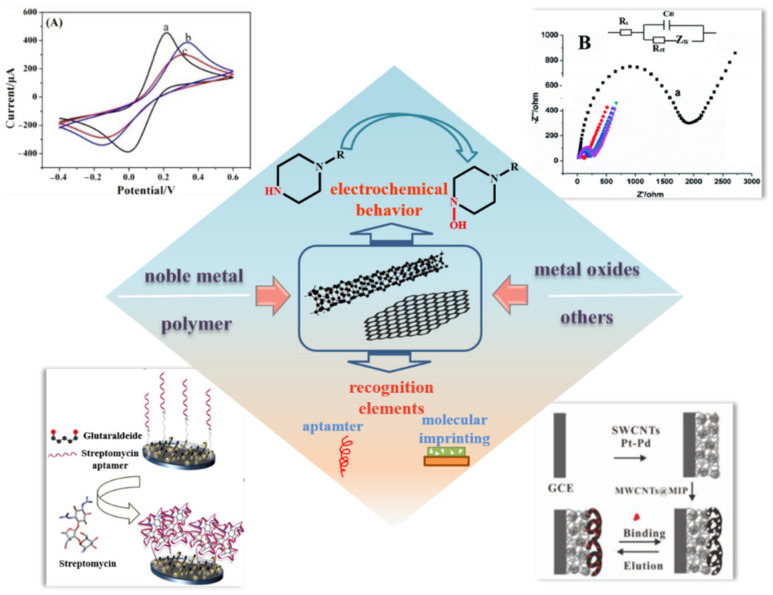
An overview of functional CNMs and their application in the detection of antibiotics.

This paper presents a comprehensive overview of reported progress, in recent years, in the electrochemical application of CNMs to detect antibiotics (Figure 1). First, we provide a brief review of the modifications of CNMs. Then, the electrochemical signal amplification of antibiotics is summarized and the application of recognition elements for the detection of antibiotics is outlined. Finally, the challenge of CNMs in antibiotic development is discussed.

## 2. Carbon-Based Nanomaterials

CNMs are considered a promising candidate for sensing antibiotic application. In order to endow CNMs with sensing capabilities, it is necessary to functionalize them with various strategies that boost the signal transmission and bring the detection targets to the surface through specific interactions [33,34,35]. Therefore, functionalized CNMs play a vital role in detecting antibiotics by electrochemical sensors. Different molecules used to modify CNMs have been selected and summarized in Table 1.

At present, various strategies have been put in place to make the CNMs functional. The intentional introduction of noble metal nanoparticles (NM NPs) is an effective method and provides great performance. Due to the more significant number of active sites on the surface of NM NPs, the conductivity can be significantly increased, the redox potential of antibiotics can be reduced, and the catalytic efficiency can be increased [36,37,38,39,40,41,54]. Ayat Mohammad-Razdari et al. reported that gold nanoparticle (Au NP)/rGO nanocomposites were prepared by electrodeposition [55]. The combination of rGO and Au NPs as a nanocomposite provides higher electrochemical activity and high electron transfer capability. In addition, NM NPs can endow excellent biocompatibility with CNMs. Mahmoud Roushani et al. reported that nanocomposite prepared from GO and functionalized with (3-aminopropyl) triethoxysilane/silver nanoparticles (Ag NPs) [56]. This nanocomposite can successfully bind the aptamer by forming a Ag–NH_2_ bond between Ag NPs and the NH_2_ group of aptamers. In addition, building bimetallic nanohybrid components that modify CNMs to detect antibiotics is of interest. Neeraj Kumar and his colleagues observed that, electrochemically, rGO modified with gold–palladium nanoparticles (Au NPs–Pd NPs–ErGO) is highly resistant to interference [42]. In their study, Au NPs could enhance electronic conductivity and Pd NPs required stability for the fabrication of a stable, rapid, and efficient voltammetric sensor. The complexes indicate high sensitivity and the efficient electrocatalytic activity that facilitated the electron transfer.

**Figure 2:**
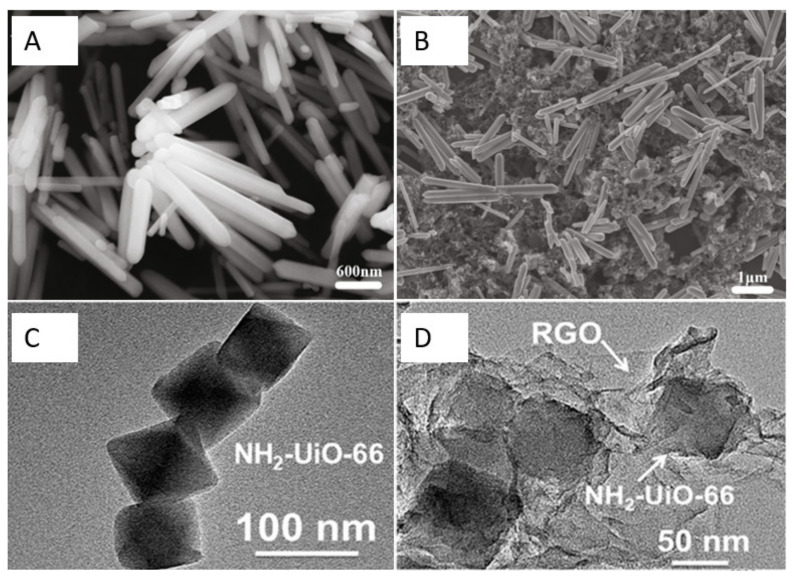
(**A**,**B**) SEM images of ZnO nanorods and Gr-ZnO nanocomposites, respectively. Adapted with permission from Ref. [44]. Copyright 2020 Elsevier. (**C**,**D**) TEM images of NH2–UiO-66 and NH2–UiO-66/RGO, respectively. Adapted with permission from Ref. [52]. Copyright 2019 ACS.

Additionally, metal oxides can endow CNMs with remarkable catalytic capabilities and fast electron transfer [43,44,45,46,47,57]. As shown in Figure 2A,B, Gr and zinc oxide nanorods (Gr-ZnO) for the detection of sulfamethoxazole were successfully synthesized [48]. The composite of Gr and ZnO can exhibit high electric conductivity and significantly improve the electron transfer rate compared to the single materials due to the synergistic effect of using them together. In addition, the decoration of CNMs by magnetic spinel ferrite nanoparticles with the chemical formula MFe_2_O_4_ (M can be an element such as Ni, Cr, and so on) can enhance electron transfer and increase sensitivity. For example, the NiFe_2_O_4_decorated rGO was successfully synthesized via a solvothermal method because of its synergistic effect. It can enhance electron transfer, speeding up the electron transfer rate and increasing sensitivity [49]. 

The combination of CNMs and polymers also led to satisfactory results in this field [49,50,51,58,59,60]. The polymelamine film on ErGO was designed using a potentiodynamic method for the determination of tetracycline [61]. The three amine groups with a triazine ring make the polymelamine useful for polymerization, and the nitrogen-rich matrix with many π-electrons can interact with the target molecules. The synergistic effect of melamine and ErGO can bolster electrochemical activity.

Thanks to the sustainable development of CNMs, researchers have been devoting themselves to developing new carbon nanocomposites to improve the detection limit and performance of electrochemical sensors for antibiotics. Xian Fang et al. reported that NH_2_-UiO-66 and rGO composites were successfully synthesized by a solvothermal method (see Figure 2C,D) [52]. In this strategy, the NH_2_-UiO-66 possesses a large surface area, and the introduction of rGO further increases the surface area of the nanocomposite and facilitates the adsorption of antibiotics. Moreover, ionic liquids are commonly used as surface modifiers because of their high conductivity, wide potential window, high viscosity, and low volatility [53]. The use of electrodes with ionic liquid associated with GO improves absorption.

## 3. Recent Reports on Antibiotic Detection by the Sensor

### 3.1. Electrochemical Behaviour of Antibiotics

The electrochemical behaviour of antibiotics is determined by the fundamental skeleton and the derived functional groups. Given the limitations of antibiotic redox behaviour, adding CNMs to electrodes could be an effective way to address this problem. A brief list of the electrochemical behaviour of commonly used antibiotics has been selected and summarized in Table 2.

#### 3.1.1. Tetracyclines

**Figure 3:**
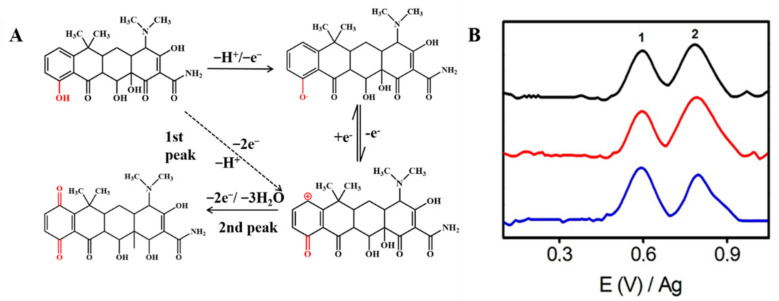
(**A**) Mechanism for the electrochemical oxidation of TET. (**B**) Adsorptive transfer stripping differential pulse voltammograms corresponding to different mixtures of tetracyclines using ErGO. The black, red, and blue lines are different concentrations of the TET antibiotic mixture. Adapted with permission from Ref. [62].

Tetracycline antibiotics, including tetracycline (TET) and oxytetracycline (OTET), have electroactive groups in their chemical structures that can be electrochemically detected [53,70,71,72]. Taking TET as an example, as shown in Figure 3A, when the potential is applied to the electrode, part of TET forms a positively charged structure which loses two electrons and a proton directly (the first peak), as the first oxide peak shows. Next, it will be combined with the strong negative oxygen in the hydrone, thereby generating a new substance that contains the structure of quinone (the second peak). The two peaks can be clearly shown in Figure 3B [62]. In this way, we can construct an electrochemical sensor with CNM composites for the detection of TET.

Due to the synergy between GO and MWCNT, the composite nanomaterial formed by GO and MWCNT increased the electroactive area of the electrode, thereby increasing the electrochemical signal and the stability of the sensor. Ademar Wong’s team reports that an electrochemical sensor based on an electrode modified with a combination of MWCNT and GO was developed for the sensitive and selective determination of TET [63]. Under the optimal conditions, the sensor showed a limit of detection (LOD) of 0.36 μM. The sensor was successfully used to detect tetracycline in river water, artificial urine, and pharmaceutical samples without any sample pretreatment.

Srinivasan Kesavan et al. designed an rGO combined with polymelamine film and modified GCE for electrochemical detection of TET [61]. There are three triazine rings on the three amino groups of melamine so that the molecules can be used for polymerization and the nitrogen-rich matrix with many π-electrons can interact with the target molecules. The results show that the current response increases linearly with the concentration from 5.0 μM to 225 μM, and the LOD is 5 μM. Moreover, the method was also used for the detection of uric acid, which shows the excellent interference resistance of the scheme. Not only polymelamine but also L-Cys can be electrodeposited on CNMs to produce a strongly adsorbed polymer film. A novel electrochemical sensor based on the Gr/L-Cys composite film modified electrode was prepared by Xuemei Sun’s team [51]. The Gr/L-Cys composite film has a uniform morphology with a diameter ranging from 50 to 100 nm, which is beneficial to the charge conduction and the adsorption of tetracycline. Under the optimized conditions, it exhibited a good linear relationship in the concentration range from 8.0 to 140 µM, and the LOD was 0.12 µM. The prepared electrode showed a practical prospect in real samples, recovering from 89.60 to 105.20%.

The use of metal peroxides also showed excellent results. An electrochemical sensor for the determination of OTET by GCE modified by Ta_2_O_5_-rGO nanocomposites was reported by Felista Magesa et al. [43]. Ta_2_O_5_-rGO composites have a large electrochemically active area that amplifies OTET oxidation signals, significantly increasing the electrochemical performance. Detection in milk samples showed that, except for a good recovery rate of 100.1–120.9%.

#### 3.1.2. Amoxicillin

**Figure 4:**
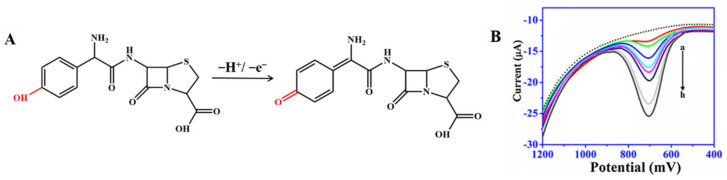
(**A**) Mechanism for the electrochemical oxidation of amoxicillin; (**B**) Square-wave voltammograms recorded for amoxicillin of different concentrations using AuNP-PdNP-ErGO/GCE. Reprinted with permission from. Adapted with permission from Ref. [42]. Copyright 2017 Elsevier.

Amoxicillin can present a weak voltammetric signal on electrodes, but CNM-modified electrodes can enhance this signal [37,45,64,65,73]. As shown in Figure 4A, the electrochemical response showed an oxidation peak, which is due to the oxidation reaction of the phenolic (–OH) substituent to the respective carbonyl group (=O) on the side chain of the molecule [71]. In the study by Behzad Rezaei and Sajjad Damiri, amoxicillin yielded a clear oxidation peak on the MWCNT-modified electrode at nearly 1.0 and 0.6 V for pH 3.0 and 7.5, respectively, and the oxidation peak current significantly increased compared with that at the bare electrode. Neeraj Kumar et al. [72] reported that AuNP-PdNP-ErGO was used to modify GCE for the determination of amoxicillin [42]. The synergy between Au and Pd nanoparticles on rGO improved sensor sensitivity and performance, thereby improving charge transfer and creating a large number of adsorption sites on the modified GCE surface. As shown in Figure 4B, the peak current of amoxicillin was found to increase with the increasing concentration of amoxicillin. Furthermore, this sensor has been developed for the individual and simultaneous determination of lomefloxacin and amoxicillin, and thereby, this shows its excellent selectivity The LOD was as low as 81 nM for lomefloxacin and 9 μM for amoxicillin. The method was successfully applied to the detection of the presence of lomefloxacin and amoxicillin in the complex matrix.

#### 3.1.3. Quinolones

**Figure 5:**
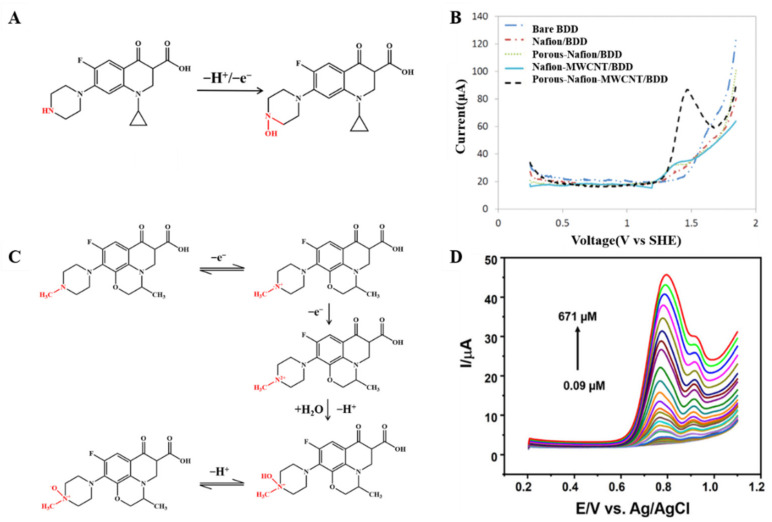
Mechanism for the electrochemical levoxidation of ciprofloxacin (**A**) and ofloxacin (**C**). (**B**) DPV curves of 50 µM ciprofloxacin on BDD, Nafion/BDD, porous-Nafion/BDD, Nafion-MWCNT/BDD, and porous-Nafion-MWCNT/BDD electrodes in 0.1 M KH_2_PO_4_ solution (pH = 4.50). Adapted with permission from Ref. [67]. Copyright 2016 ACS. (**D**) DPV response was obtained for Ag/AgVO_3_/N-rGO/SPCE by linear addition of levofloxacin in the N_2_ environment. Adapted with permission from Ref. [74]. Copyright 2021 ACS.

The redox behaviour of quinolone antibiotics allows their electrochemical study. Ciprofloxacin is a third-generation quinolone antibiotic widely used in humans and livestock. The reaction pathway of ciprofloxacin is based on the oxidation of the terminal nitrogen atom within the piperazine ring (as shown in Figure 5A) [24]. Jorge M.P.J. Garrido’s team took advantage of cyclodextrin (CD) to chemically identify the guest molecules, resulting in a novel polyaniline (PANI-β-CD/fMWCNT) that significantly improved the electron transfer rate [66]. Under optimized conditions, a linear calibration curve was obtained for ciprofloxacin in the concentration range of 10–80 mM with an LOD of 50 nM. In order to achieve rapid determination of trace antibiotics, the behaviour of heavy metals that can form stable complexes with ciprofloxacin attracted attention. Xian Fang and his team reported an electrochemical sensor for detecting ciprofloxacin using NH_2_-UiO-66 and rGO composites as working electrodes [52]. In the presence of ciprofloxacin, the oxidation current peak of Cu^2+^ decreases, apparently due to complex formation. The electrochemical sensor shows a low LOD (6.67 nM) and high sensitivity (10.86 μA/μM) based on this principle. Meanwhile, the electrochemical sensor can detect ciprofloxacin in real water samples with satisfactory recovery.

In addition, electrodes modified with nickel oxide nanoparticles (NiO NPs) have a remarkable catalytic capacity. Anderson Martin Santos et al. report a simple and highly selective electrochemical method for simultaneous determination of paracetamol and ciprofloxacin based on the binding advantages of GO and NiO NPs [46]. The sensor shows an LOD of 6.7 and 6.0 nM, respectively (see Figure 5B). The boron-doped diamond (BDD) electrode substrate was modified as the MWCNTs, which are dispersed in a porous Nafion film to develop a synergistically amplified electrochemical sensing platform for the detection of ciprofloxacin. As shown in Figure 5B, ciprofloxacin showed the highest peak at the porous-Nafion-MWCNT/BDD electrode, and DPV studies revealed an LOD of 5 nM [67].

The overall possible explanation for the electron transfer mechanism toward levofloxacin is represented in Figure 5C. Levofloxacin shows an oxidation peak variation, which is recorded for the peak produced by the piperazine moiety by a two-step condensation emitting one electron each (as shown in Figure 5D) [68,70,74]. The use of ionic liquids as modifiers on the surface of electrochemical sensors can improve compounds’ stability, sensitivity, and selectivity for oxidation and/or reduction. An electrochemical sensor based on a carbon paste electrode modified with GO and ionic liquids for the sensitive voltammetric determination of ofloxacins [53]. It shows a low LOD of 0.28 nM.

#### 3.1.4. Nitrofurans

**Figure 6:**
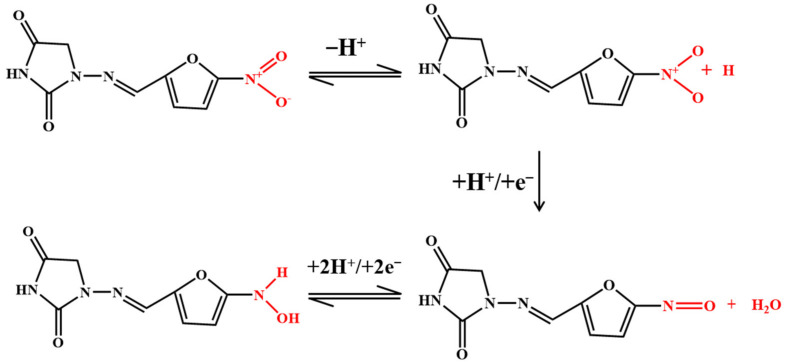
Mechanism for the electrochemical oxidation of nitrofurans.

Nitrofuran antibiotics are synthetic broad-spectrum antibiotics with 5-nitrofuran as their basic structure. The electrochemical activity of furan is related to its irreversible reduction of the nitro (–NO_2_) to nitroso intermediate, which is rapidly reduced to the corresponding hydroxylamine group (–NHOH), as shown in Figure 6 [48,69,75]. Kuo-Yuan Hwa and Tata Sanjay Kanna Sharma reported using a NiFe/MWCNTs hybrid composite as an electrocatalyst to detect nitrofurantoin. The composite has a high surface area and high electron transport, which reduces the charge-transfer resistance. The results show that the composite has good conductivity and excellent electrocatalytic activity. The LOD is 0.03 μM and the sensitivity is 11.45 μA μM^−1^cm^−2^. The NiFe_2_O_4_/rGO nanocomposites were prepared using a hydrothermal method. The high specific surface area and better electrical conductivity of graphene help to improve the signal. In addition, graphene as a carrier modified by NiFe_2_O_4_ nanoparticles can prevent any aggregation of these particles [75]. The electrochemical sensor has a linear correlation with the concentration of furazolidone in the linear range of 0.1–10.0 μM and 10.0–150.0 μM, and the LOD is 0.05 μM.

### 3.2. Application of Recognized Components in the Detection of Antibiotics

Aptamers and molecularly imprinted polymers (MIPs) were also used to detect antibiotics in order to improve binding between antibiotics and electrodes.

#### 3.2.1. Aptamer

**Figure 7:**
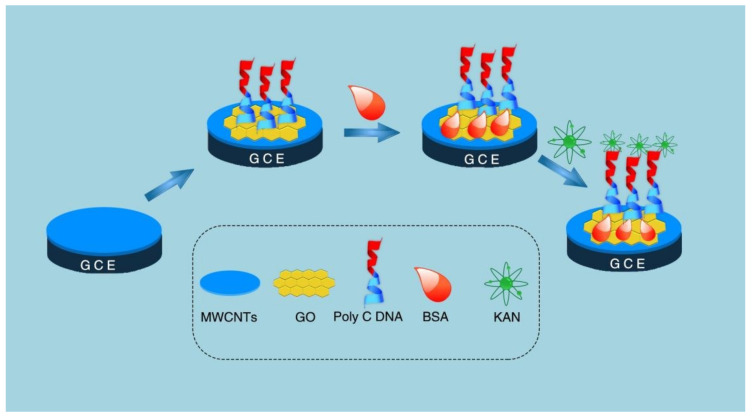
Schematic illustration of fabrication process for the DNA biosensor. Adapted with permission from Ref. [80]. Copyright 2020 Royal Society of Chemistry.

Aptamers, due to their remarkable virtues, including a wide variety of specific targets, excellent stability, and cost-effectiveness, have aroused much attention from researchers. The introduction of CNMs can significantly enhance the performance of the sensors, as shown in Table 3 [56,76,77,78,79,80,81,82]. An aptamer-based impedance method for the determination of kanamycin was reported by Azadeh Azadbakht and Amir Reza Abbasi [41]. In this report, molybdenum selenide nanoflowers (MoSe_2_) were synthesized by a hydrothermal method, and then CNTs were modified with MoSe_2_ and Au NPs as a signal amplifier. The results showed that the calibration curve was linear in the concentration range of 1 pM–0.1 nM kanamycin, and the LOD was 0.28 pM. The recovery of kanamycin in milk ranged from 96.2 to 101.5%, indicating that the method has been successfully applied to the detection of kanamycin in actual samples. Moreover, a sensor for the detection of kanamycin was prepared using the strong physical adsorption between 15-mer of poly-C and GO [82]. As shown in Figure 7, MWCNTs play a role in amplifying electrochemical signals. The designed diblock DNA includes an optimal 15-mer of poly-C and another block aptamer of kanamycin. Therefore, diblock DNA can be immobilized on GO and used for hybridization with targets. The sensor showed high sensitivity and specificity to kanamycin, with a linear range of 0.05 pM to 100 nM and an LOD of 0.0476 pM. Furthermore, the scheme has been successfully applied to the detection of milk. The results show that the scheme has a good application prospect in the detection of antibiotics in food. While indications have significant advantages, they are also limited by environmental variables such as salt concentration and pH value. Those variables also increase the complexity of their development.

#### 3.2.2. Molecular Imprinting Polymers

Molecularly imprinted polymers (MIPs) are composites of functional monomers, crosslinkers, and template molecules that can withstand a more comprehensive pH and temperature range than biomaterials. Therefore, MIPs have been widely used in the preparation of electrochemical sensors for the detection of antibiotics [40,82,83]. CNTs have been studied more extensively as carriers of MIPs. The scheme of acrylamide functionalized MWCNTs used to synthesize molecularly imprinted polymers to detect kanamycin was reported by Benzhi Liu et al. for the first time [84]. The results show that the detection range is 4 to 50 μM and the LOD is 0.68 μM. Moreover, the sensor has a good recovery rate of about 94.0–95.3% in milk and milk powder. Bimetallic particles are also used to increase their pore size to enhance the response signal. A new electrochemical sensor was composed of MWCNTs-MIP, SWCNTs, and dendritic platinum and palladium nanoparticles [85]. The ion exchange strategy was used for the first time to branch ionic liquids onto the surface of MWCNTs to prepare MIP. The function of MWCNTs is to increase the specific surface area of the electrode and increase the electrocatalytic activity of amoxicillin. The results show that the sensor obtained under the optimal conditions has a linear response to amoxicillin with a low LOD of 8.9 nM, in the range of 1.0–1000 nM. The prepared electrode was successfully applied to the determination of amoxicillin, with the recovery in honey and milk samples showing a practical prospect in real samples.

## 4. Conclusions and Prospect

This paper reviewed the latest progress in the electrochemical detection of antibiotics based on CNMs. CNM-based composites have electrocatalytic activity, good electrical conductivity, and a large specific surface area. They not only catalyze the redox reaction of antibiotics, but are also compatible with existing biometrics. Furthermore, carbon-based electrochemical sensors have been successfully developed as a rapid and sensitive method for accurate monitoring of antibiotics in food or other substances. With the rapid development of interdisciplinary cooperation, carbon-based sensors will shine in many fields, such as food, biomedicine, the environment, and so on. Recently, portable detection devices have gained popularity among researchers. Based on the excellent malleability of CNMs, the development of portable detection devices based on CNMs for the detection of antibiotics by electrochemical detection methods is also a hot topic for the future.

## Figures and Tables

**Table 1:** Different molecules are used to modify CNMs, their processing methods, and their properties.

Carbon Nanomaterials	Modification	Processing Method	Morphology	Properties	Ref.
CNTs	Ag NWs	Van der Waals interaction	Network structure	Facilitate the electron transfer.	[36]
MWCNTs	Au NPs	In situ growth	Network structure	Good sensitivity, electron-transfer rate, and surface area.	[37]
MWCNTs	Au NPs (10 nm) and cDNA	In situ growth	Tube with dark dots	High biological binding sites and good conductivity.	[38]
GO	Au NRs	Drop-casting	Rod-shaped particles, dropped-plate pieces	Good surface conductivity and conductivity.	[39]
Gr	Au NP (15 nm) and PDDA	In situ growth and electrochemical polymerization	Wrinkled flakes with bright dots	Good sensitivity and high selectivity.	[40]
CNTs	Aptamer, Au NP (68 nm) and MoSe_2_	Sonication and potentiostatic electrodeposition	Network structure with nanoflowers and spherical nanoparticles	Excellent selectivity and conductivity.	[41]
ErGO	Au NP and Pb NP	Physisorption	Wrinkled, thin film with many small spherical structures	Efficient electrocatalytic activity and good sensitivity.	[42]
ErGO	Ta_2_O_5_	Sonication and potentiostatic electrodeposition	Crumpled-like surface structure with small particles	Large electroactive surface area and better adsorption.	[43]
Gr	ZnO NRs	Sonication	The nanorods were intercalated between the sheets	High electric conductivity and sensitivity.	[44]
MWCNT	FeCr_2_O_4_ (30 nm)	In situ growth	Entangled cross-linked fibrils with small particles	Good surface conductivity.	[45]
GO	NiO NPs (24 nm)	Electrodeposition	Wrinkled sheets with multiple particles	Good surface conductivity.	[46]
MWCNT	CuO	Sonochemical	Tube wrapped in spheres	Good conductivity and catalytic activity.	[47]
rGO	NiFe_2_O_4_ (16.6 nm)	Hydrothermal method	Film with spherical-shaped particles	Good electron transfer and sensitivity.	[48]
rGO	Poly(L-cysteine) and Au NPs	Electrodeposition and in-situ growth	Wrinkled film with dark dots	Large electroactive surface area and good conductivity.	[49]
MWCNT	Poly(L-lysine)	Sonication	Tube surrounded by a blurry film	High biological binding sites and good conductivity.	[50]
Gr	Poly(L-cysteine)	Electrodeposition	Small granular structure	Good conductivity and better adsorption.	[51]
rGO	NH_2_-UiO-66 (77 nm)	Sonication	Smooth film with a regular, octahedron-like shape	Large specific surface area, high active sites, and high electrical conductivity.	[52]
Gr	ZnO	Potentiostatic electrodeposition	Smooth film with a microcluster structure	Good electro-catalytic properties and conductivity.	[53]

**Table 2:** CNM-based electrochemical sensor for antibiotics.

Atibiotics	Detection Principle	Electrode	Linear Range	LOD	Ref.
TET	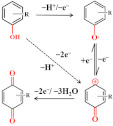	Ta_2_O_5_/ErGO/GCE	0.2–10 µM	0.095 µM	[43]
			
		L-Cys/GR/GCE	8.0–140 µM	0.12 µM	[51]
		p-Mel@ErGO/GCE	5–225 µM	2.2 µM	[61]
		ErGO/SPE	20–80 µM	12 µM	[62]
		c-MWCNTs/GO/CPE	0.02–310 µM	0.36 µM	[63]
Amoxicillin	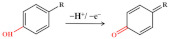	Au NPs/en-MWCNTs/SPE	0.2–10 and 10–30 µM	0.015 µM	[37]
		Au NPs/Pd NPs/ErGO/GCE	0–350 µM	9 µM	[42]
		FeCr_2_O_4_/MWCNTs/GCE	0.1–10.0 and 10.0–70.0 µM	0.05 µM	[45]
		Au NPs/Gr/Laccase/GCE	0.425–292 µM	425 nM	[64]
		MWCNTs/GCE	0.6–8.0 and 10.0–80.0 mM	0.2 µM	[65]
Ciprofloxacin	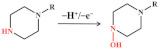	β-CD/MWCNT/GCE	10–80 µM	50 nM	[24]
		NiO NPs-GO/GCE	0.04–0.97 µM	6 nM	[46]
		NH_2_-UiO-66/RGO/GCE	0.02–1 µM	6.67 nM	[52]
		Porous-Nafion-MWCNT/BDD	0.005–10 µM	5 nM	[66]
Levofloxacin	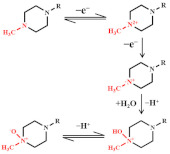	Poly (L-Cysteine)/Au NPs/rGO	0.001–100 nM	3 pM	[49]
		GO/IL/CPE	7–700 nM	0.28 nM	[53]
		Ag/AgVO3/N-rGO	0.09–670 μM	7.92 pM	[67]
Nitrofurantoin	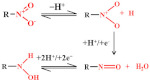	NiFe_2_O_4_/rGO/GCE	0.1–10.0 and 10.0–150.0 μM	0.05 μM	[48]
		rGO/GCE	0.001–2.0 μM and 2.0–10.0 μM	0.3 nM	[68]
		NiFe/f-MWCNT/SPCE	0.1–24.8 µM	0.03 µM	[69]

**Table 3:** CNMs-based aptasensors for antibiotics.

Antibiotics	Aptamer Sequence	Electrode	Linear Range	LOD	Ref.
TET	5′-NH_2_-(CH_2_)-CGTACGGAATTCGCTAGCCCCCCGGCAGGCCACGGCTTGGGTTGGTCCCACTGCGCGTGGATCCGAGCTCCACGTG-3′	Apt/MWCNTs/ GCE	10 nM–50 µM	5 nM	[78]
OTET	5′-SH-GGAATTCGCTAGCACGTTGACGCTGGTGCCCGGTTGTGGTGCGAGTGTTGTGTGGATCCGAGCTCCACGTG-3′	cMWCNTs/Au NPs/cDNA/Apt/ TFGE	1 × 10^−13^–1 × 10^−5^ g/mL	3.1 × 10^−14^ g/mL	[38]
	5′-NH_2_-CGTACGGAATTCGCTAGCCCCCCGGCAGGCCACGGCTTGGGTTGGTCCCACTGCGCGTGGATCCGAGCTCCACGTG-3′	Apt/GO/GCE	0.1 pM–10 µM	29 fM	[79]
	5′-SH-CGACGCACAGTCGCTGGTGCGTACCTGGTTGCCGTTGTGT-3′	Apt/Au NPs/ HRP&Au NPs/ Gr/GCE	5 × 10^−10^–2×10^−3^ g/L	4.98 × 10^−10^ g/L	[81]
Kanamycin	5′-NH_2_-(CH_2_)6-TGGGGGTTGAGGCTAAGCCGAC-3′	Apt/Au NP/ CNT/MoSe_2_/GCE	1 pM–0.1 nM and 100 nM–10 µM	0.28 pM	[41]
Streptomycin	5′-NH_2_-(CH_2_)6-TAGGGAATTCGTCGACGGATCCGGGGTCTGGTGTTCTGCTTTGTTCTGTCGGGTCGTCTGCAGGTCGACGCATGCGCCG-3′	Apt/CNT/Chi/Pd NPs/GCE	0.1–1500 nM	18 pM	[76]
	5′-NH_2_ -AGATGGGGGTTGAGGCTAAGCCGA-3′	Apt/HNP-PtCu/Gr-Thionine/GCE	5 × 10^−7^–5 × 10^−2^ μg/mL	0.42 pg/mL	[77]
Chloramphenicol	5′-NH_2_ -ACTTCAGAGAGTTGTCCCACGGTCGGCGAGTCGGTGGTAG-3′	Apt/Ag NPs/ [NH_2_-Si]-f-GO/GCE	10 pM–0.2 µM	3.3 pM	[56]

## Data Availability

Not applicable.

## References

[1] Wang H., Wang N., Wang B., Fang H., Fu C., Tang C., Jiang F., Zhou Y., He G., Zhao Q. (2016). Antibiotics detected in urines and adipogenesis in school children. Environ. Int..

[2] Mikkelsen K.H., Allin K.H., Knop F.K. (2016). Effect of antibiotics on gut microbiota, glucose metabolism and body weight regulation: A review of the literature. Diabetes Obes. Metab..

[3] Liu X., Steele J.C., Meng X.Z. (2017). Usage, residue, and human health risk of antibiotics in Chinese aquaculture: A review. Environ. Pollut..

[4] Liu S., Zhao G., Zhao H., Zhai G., Chen J., Zhao H. (2017). Antibiotics in a general population: Relations with gender, body mass index (BMI) and age and their human health risks. Sci. Total Environ..

[5] Mboya E.A., Sanga L.A., Ngocho J.S. (2018). Irrational use of antibiotics in the Moshi Municipality Northern Tanzania: A cross sectional study. Pan Afr. Med. J..

[6] Roose-Amsaleg C., Laverman A.M. (2016). Do antibiotics have environmental side-effects? Impact of synthetic antibiotics on biogeochemical processes. Environ. Sci. Pollut. Res..

[7] Nicolaou K.C., Rigol S. (2018). A brief history of antibiotics and select advances in their synthesis. J. Antibiot..

[8] Hutchings M., Truman A., Wilkinson B. (2019). Antibiotics: Past, present and future. Curr. Opin. Microbiol..

[9] Sachi S., Ferdous J., Sikder M.H., Azizul Karim Hussani S.M. (2019). Antibiotic residues in milk: Past, present, and future. J. Adv. Vet. Anim. Res..

[10] Kim C., Ryu H.D., Chung E.G., Kim Y., Lee J.K. (2018). A Review of analytical procedures for the simultaneous determination of medically important veterinary antibiotics in environmental water: Sample preparation, liquid chromatography, and mass spectrometry. J. Environ. Manag..

[11] Perez J.J., Chen C.Y. (2018). Detection of acetyltransferase modification of kanamycin, an aminoglycoside antibiotic, in bacteria using ultrahigh-performance liquid chromatography tandem mass spectrometry. Rapid Commun. Mass Spectrom..

[12] Wu Q., Peng D., Liu Q., Bakr Shabbir M.A., Sajid A., Liu Z., Wang Y., Yuan Z. (2019). A novel microbiological method in microtiter plates for screening seven kinds of widely used antibiotics residues in milk, chicken egg and honey. Front. Microbiol..

[13] Decheng S., Peilong W., Yang L., Ruiguo W., Shulin W., Zhiming X., Su Z. (2018). Simultaneous determination of antibiotics and amantadines in animal-derived feedstuffs by ultraperformance liquid chromatographic-tandem mass spectrometry. J. Chromatogr. B Anal. Technol. Biomed. Life Sci..

[14] Ezenduka E.V., Okorie-Kanu O.J., Nwanta J.A. (2019). Comparative analysis of two microbiological tests in the detection of oxytetracycline residue in chicken using ELISA as gold standard. J. Immunoass. Immunochem..

[15] Gaudin V., Rault A., Hedou C., Soumet C., Verdon E. (2017). Strategies for the screening of antibiotic residues in eggs: Comparison of the validation of the classical microbiological method with an immunobiosensor method. Food Addit. Contam. Part A Chem. Anal. Control Expo. Risk Assess..

[16] Sánchez-Hernández L., Domínguez-Vega E., Montealegre C., Castro-Puyana M., Marina M.L., Crego A.L. (2014). Potential of vancomycin for the enantiomeric resolution of FMOC-amino acids by capillary electrophoresis-ion-trap-mass spectrometry. Electrophoresis.

[17] Fu Q., Long C., Qin L., Jiang Z., Qing T., Zhang P., Feng B. (2021). Fluorescent and colorimetric dual-mode detection of tetracycline in wastewater based on heteroatoms-doped reduced state carbon dots. Environ. Pollut..

[18] Tan B., Zhao H., Du L., Gan X., Quan X. (2016). A versatile fluorescent biosensor based on target-responsive graphene oxide hydrogel for antibiotic detection. Biosens. Bioelectron..

[19] Dong X., Yan X., Li M., Liu H., Li J., Wang L., Wang K., Lu X., Wang S., He B. (2020). Ultrasensitive detection of chloramphenicol using electrochemical aptamer sensor: A mini review. Electrochem. Commun..

[20] Yue F., Li F., Kong Q., Guo Y., Sun X. (2021). Recent advances in aptamer-based sensors for aminoglycoside antibiotics detection and their applications. Sci. Total Environ..

[21] Majdinasab M., Mitsubayashi K., Marty J.L. (2019). Optical and electrochemical sensors and biosensors for the detection of quinolones. Trends Biotechnol..

[22] Sun Y., Zhao J., Liang L. (2021). Recent development of antibiotic detection in food and environment: The combination of sensors and nanomaterials. Microchim. Acta.

[23] Kharewal T., Verma N., Gahlaut A., Hooda V. (2020). Biosensors for penicillin quantification: A comprehensive review. Biotechnol. Lett..

[24] Rudnicki K., Sipa K., Brycht M., Borgul P., Skrzypek S., Poltorak L. (2020). Electrochemical sensing of fluoroquinolone antibiotics. TrAC.

[25] Gaudin V. (2017). Advances in biosensor development for the screening of antibiotic residues in food products of animal origin—A Comprehensive Review. Biosens. Bioelectron..

[26] Pollap A., Kochana J. (2019). Electrochemical immunosensors for antibiotic detection. Biosensors.

[27] Sainz-Urruela C., Vera-López S., San Andrés M.P., Díez-pascual A.M. (2021). Graphene-based sensors for the detection of bioactive compounds: A review. Int. J. Mol. Sci..

[28] Tîlmaciu C.M., Morris M.C. (2015). Carbon Nanotube Biosensors. Front. Chem..

[29] Joshi A., Kim K.H. (2020). Recent Advances in nanomaterial-based electrochemical detection of antibiotics: Challenges and future perspectives. Biosens. Bioelectron..

[30] Teradal N.L., Jelinek R. (2017). Carbon nanomaterials in biological studies and biomedicine. Adv. Healthc. Mater..

[31] Ma X., Li X., Zhang W., Meng F., Wang X., Qin Y., Zhang M. (2021). Carbon-Based Nanocomposite Smart Sensors for the Rapid Detection of Mycotoxins. Nanomaterials.

[32] Meng F., Qin Y., Zhang W., Chen F., Zheng L., Xing J., Aihaiti A., Zhang M. (2022). Amplified electrochemical sensor employing Ag NPs functionalized graphene paper electrode for high sensitive analysis of Sudan, I. Food Chem..

[33] Baptista F.R., Belhout S.A., Giordani S., Quinn S.J. (2015). Recent developments in carbon nanomaterial sensors. Chem. Soc. Rev..

[34] Sanati A., Jalali M., Raeissi K., Karimzadeh F., Kharaziha M., Mahshid S.S., Mahshid S. (2019). A review on recent advancements in electrochemical biosensing using carbonaceous nanomaterials. Microchim. Acta.

[35] Zhang M., Hou C., Halder A., Ulstrup J., Chi Q. (2017). Interlocked graphene-Prussian blue hybrid composites enable multifunctional electrochemical applications. Biosens. Bioelectron..

[36] Zhu Y., Li X., Xu Y., Wu L., Yu A., Lai G., Wei Q., Chi H., Jiang N., Fu L. (2021). Intertwined carbon nanotubes and Ag nanowires constructed by simple solution blending as sensitive and stable chloramphenicol sensors. Sensors.

[37] Muhammad A., Yusof N.A., Hajian R., Abdullah J. (2016). Construction of an electrochemical sensor based on carbon nanotubes/gold nanoparticles for trace determination of amoxicillin in bovine milk. Sensors.

[38] He B., Wang L., Dong X., Yan X., Li M., Yan S., Yan D. (2019). Aptamer-based thin film gold electrode modified with gold nanoparticles and carboxylated multi-walled carbon nanotubes for detecting oxytetracycline in chicken samples. Food Chem..

[39] Golkarieh A.M., Nasirizadeh N., Jahanmardi R. (2021). Fabrication of an electrochemical sensor with Au nanorods-graphene oxide hybrid nanocomposites for in situ measurement of cloxacillin. Mater. Sci. Eng. C.

[40] Wang F., Zhu L., Zhang J. (2014). Electrochemical sensor for levofloxacin based on molecularly imprinted polypyrrole-graphene-gold nanoparticles modified electrode. Sens. Actuators B Chem..

[41] Azadbakht A., Abbasi A.R. (2019). Impedimetric aptasensor for kanamycin by using carbon nanotubes modified with MoSe_2_ nanoflowers and gold nanoparticles as signal amplifiers. Microchim. Acta.

[42] Kumar N., Goyal R.N. (2017). Gold-palladium nanoparticles aided electrochemically reduced graphene oxide sensor for the simultaneous estimation of lomefloxacin and amoxicillin. Sens. Actuators B Chem..

[43] Magesa F., Wu Y., Dong S., Tian Y., Li G., Vianney J.M., Buza J., Liu J., He Q. (2020). Electrochemical sensing fabricated with Ta_2_O_5_ nanoparticle-electrochemically reduced graphene oxide nanocomposite for the detection of oxytetracycline. Biomolecules.

[44] Yue X., Li Z., Zhao S. (2020). A new electrochemical sensor for simultaneous detection of sulfamethoxazole and trimethoprim antibiotics based on graphene and ZnO nanorods modified glassy carbon electrode. Microchem. J..

[45] Ensafi A.A., Allafchian A.R., Rezaei B. (2012). Multiwall carbon nanotubes decorated with FeCr_2_O_4_, a new selective electrochemical sensor for amoxicillin determination. J. Nanoparticle Res..

[46] Martin Santos A., Wong A., Almeida A.A., Fatibello-Filho O. (2017). Simultaneous determination of paracetamol and ciprofloxacin in biological fluid samples using a glassy carbon electrode modified with graphene oxide and nickel oxide nanoparticles. Talanta.

[47] Chen T.W., Rajaji U., Chen S.M., Muthumariyappan A., al Mogren M.M., Jothi Ramalingam R., Hochlaf M. (2019). Facile synthesis of copper(II) oxide nanospheres covered on functionalized multiwalled carbon nanotubes modified electrode as rapid electrochemical sensing platform for super-sensitive detection of antibiotic. Ultrason. Sonochem..

[48] Ensafi A.A., Zandi-Atashbar N., Gorgabi-Khorzoughi M., Rezaei B. (2019). Nickel-ferrite oxide decorated on reduced graphene oxide, an efficient and selective electrochemical sensor for detection of furazolidone. IEEE Sens. J..

[49] Ghanbari M.H., Khoshroo A., Sobati H., Ganjali M.R., Rahimi-Nasrabadi M., Ahmadi F. (2019). An electrochemical sensor based on poly (L-Cysteine)@AuNPs @ reduced graphene oxide nanocomposite for determination of levofloxacin. Microchem. J..

[50] He Z., Zang S., Liu Y., He Y., Lei H. (2015). A multi-walled carbon nanotubes-poly(L-lysine) modified enantioselective immunosensor for ofloxacin by using multi-enzyme-labeled gold nanoflower as signal enhancer. Biosens. Bioelectron..

[51] Sun X.-M., Ji Z., Xiong M.-X., Chen W. (2017). The electrochemical sensor for the determination of tetracycline based on graphene /L-cysteine composite film. J. Electrochem. Soc..

[52] Fang X., Chen X., Liu Y., Li Q., Zeng Z., Maiyalagan T., Mao S. (2019). Nanocomposites of Zr(IV)-based metal-organic frameworks and reduced graphene oxide for electrochemically sensing ciprofloxacin in water. ACS Appl. Nano Mater..

[53] Wong A., Silva T.A., Vicentini F.C., Fatibello-Filho O. (2016). Electrochemical sensor based on graphene oxide and ionic liquid for ofloxacin determination at nanomolar levels. Talanta.

[54] Kirchner E.M., Hirsch T. (2020). Recent developments in carbon-based two-dimensional materials: Synthesis and modification aspects for electrochemical sensors. Microchim. Acta.

[55] Mohammad-Razdari A., Ghasemi-Varnamkhasti M., Izadi Z., Ensafi A.A., Rostami S., Siadat M. (2019). An impedimetric aptasensor for ultrasensitive detection of Penicillin G based on the use of reduced graphene oxide and gold nanoparticles. Microchim. Acta.

[56] Roushani M., Rahmati Z., Farokhi S., Hoseini S.J., Fath R.H. (2020). The development of an electrochemical nanoaptasensor to sensing chloramphenicol using a nanocomposite consisting of graphene oxide functionalized with (3-aminopropyl) triethoxysilane and silver nanoparticles. Mater. Sci. Eng. C.

[57] Gao C., Guo Z., Liu J.H., Huang X.J. (2012). The new age of carbon nanotubes: An updated review of functionalized carbon nanotubes in electrochemical sensors. Nanoscale.

[58] Hatamluyi B., Lorestani F., Es’haghi Z. (2018). Au/Pd@rGO nanocomposite decorated with poly (L-cysteine) as a probe for simultaneous sensitive electrochemical determination of anticancer drugs, Ifosfamide and Etoposide. Biosens. Bioelectron..

[59] Kuilla T., Bhadra S., Yao D., Kim N.H., Bose S., Lee J.H. (2010). Recent advances in graphene based polymer composites. Prog. Polym. Sci..

[60] Arvand M., Sanayeei M., Hemmati S. (2018). Label-free electrochemical DNA biosensor for guanine and adenine by ds-DNA/poly(L-cysteine)/Fe_3_O_4_ nanoparticles-graphene oxide nanocomposite modified electrode. Biosens. Bioelectron..

[61] Kesavan S., Kumar D.R., Lee Y.R., Shim J.J. (2017). Determination of tetracycline in the presence of major interference in human urine samples using polymelamine/electrochemically reduced graphene oxide modified electrode. Sens. Actuators B Chem..

[62] Lorenzetti A.S., Sierra T., Domini C.E., Lista A.G., Crevillen A.G., Escarpa A. (2020). Electrochemically reduced graphene oxide-based screen-printed electrodes for total tetracycline determination by adsorptive transfer stripping differential pulse voltammetry. Sensors.

[63] Wong A., Scontri M., Materon E.M., Lanza M.R.V., Sotomayor M.D.P.T. (2015). Development and application of an electrochemical sensor modified with multi-walled carbon nanotubes and graphene oxide for the sensitive and selective detection of tetracycline. J. Electroanal. Chem..

[64] Osikoya A.O., Opoku F., Govender P.P. (2021). Electrochemical detection of amoxicillin on 2D graphene-gold nanoparticle-lacasse bio-interfaces: Combined experimental and theoretical study. Chem. Phys. Lett..

[65] Rezaei B., Damiri S. (2009). Electrochemistry and adsorptive stripping voltammetric determination of amoxicillin on a multiwalled carbon nanotubes modified glassy carbon electrode. Electroanalysis.

[66] Garrido J.M.P.J., Melle-Franco M., Strutyński K., Borges F., Brett C.M.A., Garrido E.M.P.J. (2017). β–Cyclodextrin carbon nanotube-enhanced sensor for ciprofloxacin detection. J. Environ. Sci. Health Part A.

[67] Gayen P., Chaplin B.P. (2016). Selective electrochemical detection of ciprofloxacin with a porous nafion/multiwalled carbon nanotube composite film electrode. ACS Appl. Mater. Interfaces.

[68] Huang K.J., Liu X., Xie W.Z., Yuan H.X. (2008). Electrochemical behavior and voltammetric determination of norfloxacin at glassy carbon electrode modified with multi walled carbon nanotubes/nafion. Colloids Surf. B Biointerfaces.

[69] Shahrokhian S., Naderi L., Ghalkhani M. (2016). Modified glassy carbon electrodes based on carbon nanostructures for ultrasensitive electrochemical determination of furazolidone. Mater. Sci. Eng. C.

[70] Si X., Wei Y., Wang C., Li L., Ding Y. (2018). A sensitive electrochemical sensor for ofloxacin based on a graphene/zinc oxide composite film. Anal. Methods.

[71] Raykova M.R., Corrigan D.K., Holdsworth M., Henriquez F.L., Ward A.C. (2021). Emerging electrochemical sensors for real-time detection of tetracyclines in milk. Biosensors.

[72] Yáñez-Sedeño P., Pedrero M., Campuzano S., Pingarrón J.M. (2021). Electrocatalytic (bio)platforms for the determination of tetracyclines. J. Solid State Electrochem..

[73] Hrioua A., Loudiki A., Farahi A., Bakasse M., Lahrich S., Saqrane S., EL Mhammedi M.A. (2021). Recent advances in electrochemical sensors for amoxicillin detection in biological and environmental samples. Bioelectrochemistry.

[74] Sharma T.S.K., Hwa K.Y. (2021). Facile synthesis of Ag/AgVO_3_/N-RGO hybrid nanocomposites for electrochemical detection of levofloxacin for complex biological samples using screen-printed carbon paste electrodes. Inorg. Chem..

[75] Hwa K.Y., Sharma T.S.K. (2020). Nano assembly of NiFe spheres anchored on f-MWCNT for electrocatalytic reduction and sensing of nitrofurantoin in biological samples. Sci. Rep..

[76] Aghajari R., Azadbakht A. (2018). Amplified detection of streptomycin using aptamer-conjugated palladium nanoparticles decorated on chitosan-carbon nanotube. Anal. Biochem..

[77] Qin X., Yin Y., Yu H., Guo W., Pei M. (2016). A novel signal amplification strategy of an electrochemical aptasensor for kanamycin, based on thionine functionalized graphene and hierarchical nanoporous PtCu. Biosens. Bioelectron..

[78] Zhou L., Li D.J., Gai L., Wang J.P., Li Y.B. (2012). Electrochemical aptasensor for the detection of tetracycline with multi-walled carbon nanotubes amplification. Sens. Actuators B Chem..

[79] Benvidi A., Tezerjani M.D., Moshtaghiun S.M., Mazloum-Ardakani M. (2016). An aptasensor for tetracycline using a glassy carbon modified with nanosheets of graphene oxide. Microchim. Acta.

[80] He X., Han H., Shi W., Dong J., Lu X., Yang W., Lu X. (2020). A label-free electrochemical DNA biosensor for kanamycin detection based on diblock DNA with poly-cytosine as a high affinity anchor on graphene oxide. Anal. Methods.

[81] Liu S., Wang Y., Xu W., Leng X., Wang H., Guo Y., Huang J. (2017). A novel sandwich-type electrochemical aptasensor based on GR-3D Au and aptamer-AuNPs-HRP for sensitive detection of oxytetracycline. Biosens. Bioelectron..

[82] Işık D., Şahin S., Caglayan M.O., Üstündağ Z. (2021). Electrochemical impedimetric detection of kanamycin using molecular imprinting for food safety. Microchem. J..

[83] Shi X., Zuo Y., Jia X., Wu X., Jing N., Wen B., Mi X. (2020). A novel molecularly imprinted sensor based on gold nanoparticles/reduced graphene oxide/single-walled carbon nanotubes nanocomposite for the detection of pefloxacin. Int. J. Electrochem. Sci..

[84] Liu B., Ma Y., Zhou F., Wang Q., Liu G. (2020). Voltammetric determination of sulfadiazine based on molecular imprinted electrochemical sensor. Int. J. Electrochem. Sci..

[85] Yang G., Zhao F. (2015). Molecularly imprinted polymer grown on multiwalled carbon nanotube surface for the sensitive electrochemical determination of amoxicillin. Electrochim. Acta.

